# Supplementary material for: Fluctuations of psychological states on Twitter before and during COVID-19
Source: PLoS One. 2022 Dec 14;17(12):e0278018. doi: 10.1371/journal.pone.0278018 (PMC9750014; doi:10.1371/journal.pone.0278018)
Supplement: S1 Table — Note. NegEmo = Negative emotion; PosEmo = positive emotion; Linguistic Inquiry and Word Count (LIWC) scores represent percentages of total in-category words within a given text. (DOCX) [file pone.0278018.s001.docx]

**Table S1**

Mean monthly LIWC scores for tweets from London during 2020

|  | **January (N=77096)** | **February (N=75157)** | **March (N=110310)** | **April (N=122930)** | **May (N=120382)** | **June (N=113370)** | **July (N=109345)** | **August (N=104091)** | **September (N=106678)** | **October (N=121889)** | **November (N=132729)** | **December (N=120437)** | **2020 Total (N=1314414)** |
| --- | --- | --- | --- | --- | --- | --- | --- | --- | --- | --- | --- | --- | --- |
| **Sadness** |  |  |  |  |  |  |  |  |  |  |  |  |  |
| Mean (SD) | 0.48 (3.53) | 0.46 (3.27) | 0.54 (3.48) | 0.54 (3.49) | 0.51 (3.43) | 0.50 (3.52) | 0.47 (3.10) | 0.48 (3.32) | 0.47 (3.23) | 0.49 (3.38) | 0.56 (4.12) | 0.50 (3.39) | 0.50 (3.46) |
| **Anxiety** |  |  |  |  |  |  |  |  |  |  |  |  |  |
| Mean (SD) | 0.25 (2.38) | 0.25 (2.23) | 0.32 (2.57) | 0.26 (2.37) | 0.28 (2.48) | 0.29 (2.46) | 0.27 (2.35) | 0.27 (2.47) | 0.29 (2.52) | 0.28 (2.40) | 0.29 (2.58) | 0.27 (2.43) | 0.28 (2.45) |
| **Anger** |  |  |  |  |  |  |  |  |  |  |  |  |  |
| Mean (SD) | 0.74 (4.56) | 0.69 (4.33) | 0.67 (4.45) | 0.67 (4.35) | 0.75 (4.49) | 0.81 (4.39) | 0.77 (4.53) | 0.75 (4.49) | 0.77 (4.61) | 0.77 (4.64) | 0.79 (4.62) | 0.75 (4.48) | 0.75 (4.50) |
| **NegEmo** |  |  |  |  |  |  |  |  |  |  |  |  |  |
| Mean (SD) | 2.11 (7.47) | 2.03 (7.11) | 2.17 (7.36) | 2.11 (7.27) | 2.21 (7.39) | 2.32 (7.51) | 2.23 (7.39) | 2.18 (7.34) | 2.20 (7.47) | 2.21 (7.38) | 2.31 (7.83) | 2.16 (7.32) | 2.19 (7.42) |
| **PosEmo** |  |  |  |  |  |  |  |  |  |  |  |  |  |
| Mean (SD) | 7.54 (14.49) | 7.66 (14.50) | 7.51 (14.53) | 7.88 (15.12) | 7.31 (14.43) | 7.34 (14.55) | 7.36 (14.42) | 7.30 (14.62) | 7.17 (14.36) | 7.36 (14.77) | 7.18 (14.75) | 7.55 (14.75) | 7.42 (14.63) |
| **Work** |  |  |  |  |  |  |  |  |  |  |  |  |  |
| Mean (SD) | 2.15 (5.69) | 2.22 (5.70) | 2.24 (5.71) | 2.02 (5.55) | 2.04 (5.50) | 2.12 (5.66) | 2.08 (5.52) | 2.10 (5.73) | 2.18 (5.75) | 2.13 (5.76) | 2.03 (5.59) | 1.88 (5.48) | 2.09 (5.63) |
| **Leisure** |  |  |  |  |  |  |  |  |  |  |  |  |  |
| Mean (SD) | 1.79 (5.24) | 1.78 (5.29) | 1.71 (5.17) | 1.67 (5.10) | 1.64 (5.03) | 1.56 (4.91) | 1.72 (5.54) | 1.65 (5.19) | 1.61 (5.11) | 1.59 (5.06) | 1.55 (5.08) | 1.63 (5.03) | 1.65 (5.14) |
| **Home** |  |  |  |  |  |  |  |  |  |  |  |  |  |
| Mean (SD) | 0.32 (2.07) | 0.36 (2.42) | 0.46 (2.35) | 0.45 (2.46) | 0.39 (2.25) | 0.34 (2.25) | 0.34 (2.22) | 0.35 (2.37) | 0.33 (2.16) | 0.33 (2.32) | 0.32 (2.14) | 0.34 (2.17) | 0.36 (2.27) |
| **Health** |  |  |  |  |  |  |  |  |  |  |  |  |  |
| Mean (SD) | 0.54 (3.18) | 0.52 (2.90) | 0.69 (3.19) | 0.65 (3.19) | 0.62 (3.31) | 0.58 (3.15) | 0.54 (2.99) | 0.54 (3.04) | 0.56 (3.12) | 0.55 (3.06) | 0.51 (2.92) | 0.54 (3.02) | 0.57 (3.10) |

Note*.* NegEmo = Negative emotion; PosEmo = positive emotion; Linguistic Inquiry and Word Count (LIWC) scores represent percentages of total in-category words within a given text.
