# Supplementary material for: Fluctuations of psychological states on Twitter before and during COVID-19
Source: PLoS One. 2022 Dec 14;17(12):e0278018. doi: 10.1371/journal.pone.0278018 (PMC9750014; doi:10.1371/journal.pone.0278018)
Supplement: S2 Table — Note. NegEmo = Negative emotion; PosEmo = positive emotion; Linguistic Inquiry and Word Count (LIWC) scores represent percentages of total in-category words within a given text. (DOCX) [file pone.0278018.s002.docx]

**Table S2**

Mean monthly LIWC scores for tweets from London during 2019

|  | **January (N=53161)** | **February (N=50656)** | **March (N=61001)** | **April (N=58215)** | **May (N=64739)** | **June (N=63767)** | **July (N=66308)** | **August (N=64058)** | **September (N=66587)** | **October (N=70762)** | **November (N=70014)** | **December (N=72902)** | **2019 Total (N=762170)** |
| --- | --- | --- | --- | --- | --- | --- | --- | --- | --- | --- | --- | --- | --- |
| **Sadness** |  |  |  |  |  |  |  |  |  |  |  |  |  |
| Mean (SD) | 0.40 (2.84) | 0.44 (3.19) | 0.41 (2.87) | 0.42 (2.98) | 0.42 (3.26) | 0.44 (3.18) | 0.52 (4.24) | 0.50 (4.00) | 0.45 (3.42) | 0.45 (3.20) | 0.41 (2.90) | 0.48 (3.45) | 0.45 (3.33) |
| **Anxiety** |  |  |  |  |  |  |  |  |  |  |  |  |  |
| Mean (SD) | 0.26 (2.52) | 0.25 (2.57) | 0.25 (2.15) | 0.22 (2.18) | 0.23 (2.19) | 0.25 (2.56) | 0.25 (2.27) | 0.23 (2.07) | 0.24 (2.20) | 0.26 (2.56) | 0.26 (2.50) | 0.27 (2.48) | 0.25 (2.36) |
| **Anger** |  |  |  |  |  |  |  |  |  |  |  |  |  |
| Mean (SD) | 0.66 (4.28) | 0.64 (4.02) | 0.65 (4.01) | 0.63 (4.12) | 0.64 (4.24) | 0.66 (4.14) | 0.69 (4.43) | 0.73 (4.42) | 0.64 (4.11) | 0.67 (4.24) | 0.71 (4.56) | 0.76 (4.46) | 0.68 (4.27) |
| **NegEmo** |  |  |  |  |  |  |  |  |  |  |  |  |  |
| Mean (SD) | 1.91 (6.94) | 1.90 (6.82) | 1.87 (6.56) | 1.86 (6.78) | 1.86 (7.02) | 1.94 (7.02) | 2.04 (7.59) | 2.10 (7.53) | 1.93 (6.95) | 1.97 (6.97) | 1.96 (7.05) | 2.16 (7.46) | 1.96 (7.08) |
| **PosEmo** |  |  |  |  |  |  |  |  |  |  |  |  |  |
| Mean (SD) | 7.59 (14.08) | 7.62 (13.85) | 7.91 (14.38) | 7.92 (14.51) | 7.95 (14.54) | 7.72 (14.45) | 7.96 (15.30) | 7.63 (14.65) | 7.80 (14.66) | 7.86 (14.73) | 7.84 (14.69) | 7.86 (14.61) | 7.81 (14.57) |
| **Work** |  |  |  |  |  |  |  |  |  |  |  |  |  |
| Mean (SD) | 2.35 (5.93) | 2.41 (5.87) | 2.44 (5.99) | 2.27 (5.74) | 2.29 (5.89) | 2.23 (5.72) | 2.20 (5.95) | 2.03 (5.47) | 2.26 (5.84) | 2.25 (5.89) | 2.32 (5.78) | 2.03 (5.63) | 2.25 (5.81) |
| **Leisure** |  |  |  |  |  |  |  |  |  |  |  |  |  |
| Mean (SD) | 1.96 (5.31) | 1.94 (5.39) | 1.92 (5.33) | 2.00 (5.55) | 1.97 (5.48) | 1.93 (5.53) | 1.87 (5.48) | 1.92 (5.60) | 1.77 (5.27) | 1.81 (5.33) | 1.79 (5.23) | 1.77 (5.19) | 1.88 (5.39) |
| **Home** |  |  |  |  |  |  |  |  |  |  |  |  |  |
| Mean (SD) | 0.33 (2.08) | 0.35 (2.19) | 0.32 (2.10) | 0.38 (2.50) | 0.34 (2.21) | 0.36 (2.33) | 0.36 (2.30) | 0.35 (2.25) | 0.32 (2.10) | 0.33 (2.14) | 0.32 (2.13) | 0.36 (2.30) | 0.34 (2.22) |
| **Health** |  |  |  |  |  |  |  |  |  |  |  |  |  |
| Mean (SD) | 0.55 (3.05) | 0.52 (2.74) | 0.50 (2.80) | 0.51 (2.80) | 0.53 (3.04) | 0.51 (2.94) | 0.51 (3.01) | 0.52 (2.91) | 0.52 (2.90) | 0.53 (3.04) | 0.49 (2.79) | 0.48 (2.86) | 0.51 (2.91) |

Note*.* NegEmo = Negative emotion; PosEmo = positive emotion; Linguistic Inquiry and Word Count (LIWC) scores represent percentages of total in-category words within a given text.
