# Supplementary material for: Fluctuations of psychological states on Twitter before and during COVID-19
Source: PLoS One. 2022 Dec 14;17(12):e0278018. doi: 10.1371/journal.pone.0278018 (PMC9750014; doi:10.1371/journal.pone.0278018)
Supplement: S3 Table — Note. NegEmo = Negative emotion; PosEmo = positive emotion; Linguistic Inquiry and Word Count (LIWC) scores represent percentages of total in-category words within a given text. (DOCX) [file pone.0278018.s003.docx]

**Table S3**

Mean monthly LIWC scores for tweets from New York during 2020

|  | **January (N=52536)** | **February (N=51061)** | **March (N=69577)** | **April (N=72410)** | **May (N=67907)** | **June (N=68592)** | **July (N=75664)** | **August (N=78818)** | **September (N=79959)** | **October (N=89418)** | **November (N=95644)** | **December (N=85340)** | **2020 Total (N=886926)** |
| --- | --- | --- | --- | --- | --- | --- | --- | --- | --- | --- | --- | --- | --- |
| **Sadness** |  |  |  |  |  |  |  |  |  |  |  |  |  |
| Mean (SD) | 0.50 (3.57) | 0.45 (3.25) | 0.49 (3.41) | 0.53 (3.49) | 0.50 (3.50) | 0.49 (3.41) | 0.50 (3.47) | 0.52 (3.53) | 0.50 (3.46) | 0.51 (3.55) | 0.56 (3.81) | 0.52 (3.63) | 0.51 (3.53) |
| **Anxiety** |  |  |  |  |  |  |  |  |  |  |  |  |  |
| Mean (SD) | 0.23 (2.31) | 0.25 (2.32) | 0.29 (2.30) | 0.25 (2.42) | 0.24 (2.23) | 0.25 (2.24) | 0.24 (2.27) | 0.26 (2.40) | 0.25 (2.12) | 0.26 (2.42) | 0.26 (2.45) | 0.23 (2.26) | 0.25 (2.32) |
| **Anger** |  |  |  |  |  |  |  |  |  |  |  |  |  |
| Mean (SD) | 0.97 (5.22) | 0.95 (4.87) | 0.89 (4.83) | 0.88 (4.89) | 1.02 (5.26) | 1.19 (5.47) | 0.99 (5.01) | 1.01 (5.26) | 1.05 (5.55) | 1.02 (5.14) | 1.04 (5.52) | 0.98 (5.34) | 1.00 (5.22) |
| **NegEmo** |  |  |  |  |  |  |  |  |  |  |  |  |  |
| Mean (SD) | 2.41 (8.11) | 2.29 (7.44) | 2.35 (7.55) | 2.33 (7.70) | 2.46 (8.03) | 2.66 (8.03) | 2.47 (7.80) | 2.53 (8.12) | 2.57 (8.26) | 2.56 (8.18) | 2.63 (8.61) | 2.50 (8.40) | 2.49 (8.07) |
| **PosEmo** |  |  |  |  |  |  |  |  |  |  |  |  |  |
| Mean (SD) | 6.84 (14.34) | 6.86 (14.37) | 6.82 (14.39) | 7.06 (14.63) | 6.81 (14.41) | 6.65 (14.35) | 6.63 (14.20) | 6.79 (14.64) | 6.78 (14.45) | 6.97 (15.05) | 7.41 (15.55) | 7.43 (15.41) | 6.94 (14.71) |
| **Work** |  |  |  |  |  |  |  |  |  |  |  |  |  |
| Mean (SD) | 2.04 (5.58) | 2.41 (6.32) | 2.34 (5.84) | 2.21 (5.66) | 2.15 (5.77) | 2.24 (5.75) | 2.18 (5.74) | 2.08 (5.66) | 2.09 (5.71) | 1.99 (5.64) | 1.97 (5.65) | 1.91 (5.58) | 2.12 (5.73) |
| **Leisure** |  |  |  |  |  |  |  |  |  |  |  |  |  |
| Mean (SD) | 1.96 (5.66) | 1.83 (5.52) | 1.63 (5.22) | 1.60 (5.23) | 1.63 (5.25) | 1.44 (4.86) | 1.64 (5.41) | 1.58 (5.10) | 1.64 (5.12) | 1.54 (5.10) | 1.53 (5.10) | 1.76 (5.53) | 1.63 (5.24) |
| **Home** |  |  |  |  |  |  |  |  |  |  |  |  |  |
| Mean (SD) | 0.39 (2.77) | 0.37 (2.65) | 0.47 (2.56) | 0.46 (2.72) | 0.39 (2.43) | 0.33 (2.23) | 0.35 (2.25) | 0.35 (2.35) | 0.33 (2.29) | 0.33 (2.24) | 0.32 (2.07) | 0.35 (2.21) | 0.37 (2.38) |
| **Health** |  |  |  |  |  |  |  |  |  |  |  |  |  |
| Mean (SD) | 0.60 (3.27) | 0.57 (2.95) | 0.75 (3.49) | 0.66 (3.25) | 0.65 (3.33) | 0.60 (3.14) | 0.58 (3.22) | 0.55 (3.31) | 0.55 (3.14) | 0.55 (3.17) | 0.50 (3.09) | 0.54 (3.37) | 0.59 (3.23) |

Note*.* NegEmo = Negative emotion; PosEmo = positive emotion; Linguistic Inquiry and Word Count (LIWC) scores represent percentages of total in-category words within a given text.
