# Supplementary material for: Fluctuations of psychological states on Twitter before and during COVID-19
Source: PLoS One. 2022 Dec 14;17(12):e0278018. doi: 10.1371/journal.pone.0278018 (PMC9750014; doi:10.1371/journal.pone.0278018)
Supplement: S4 Table — Note. NegEmo = Negative emotion; PosEmo = positive emotion; Linguistic Inquiry and Word Count (LIWC) scores represent percentages of total in-category words within a given text. (DOCX) [file pone.0278018.s004.docx]

**Table S4**

Mean monthly LIWC scores for tweets from New York during 2019

|  | **January (N=32811)** | **February (N=32336)** | **March (N=35509)** | **April (N=35993)** | **May (N=35888)** | **June (N=35491)** | **July (N=37416)** | **August (N=39789)** | **September (N=41442)** | **October (N=46762)** | **November (N=46444)** | **December (N=47532)** | **2019 Total (N=467413)** |
| --- | --- | --- | --- | --- | --- | --- | --- | --- | --- | --- | --- | --- | --- |
| **Sadness** |  |  |  |  |  |  |  |  |  |  |  |  |  |
| Mean (SD) | 0.44 (3.30) | 0.45 (3.06) | 0.44 (3.21) | 0.47 (3.54) | 0.44 (3.30) | 0.47 (3.33) | 0.43 (3.12) | 0.44 (3.38) | 0.44 (3.09) | 0.42 (3.13) | 0.42 (3.31) | 0.44 (3.22) | 0.44 (3.25) |
| **Anxiety** |  |  |  |  |  |  |  |  |  |  |  |  |  |
| Mean (SD) | 0.22 (2.05) | 0.22 (2.04) | 0.24 (2.26) | 0.24 (2.64) | 0.23 (2.24) | 0.22 (2.31) | 0.27 (2.44) | 0.25 (2.51) | 0.24 (2.25) | 0.26 (2.38) | 0.23 (2.24) | 0.21 (2.32) | 0.23 (2.32) |
| **Anger** |  |  |  |  |  |  |  |  |  |  |  |  |  |
| Mean (SD) | 0.93 (5.06) | 0.84 (4.59) | 0.82 (4.42) | 0.88 (5.01) | 0.84 (4.56) | 0.91 (5.00) | 0.92 (4.92) | 0.94 (4.96) | 0.94 (5.17) | 0.91 (4.90) | 0.92 (4.92) | 0.97 (5.06) | 0.90 (4.90) |
| **NegEmo** |  |  |  |  |  |  |  |  |  |  |  |  |  |
| Mean (SD) | 2.27 (7.71) | 2.14 (7.20) | 2.15 (7.22) | 2.20 (7.71) | 2.12 (7.34) | 2.29 (8.01) | 2.34 (7.92) | 2.29 (7.74) | 2.23 (7.45) | 2.28 (7.61) | 2.17 (7.38) | 2.26 (7.56) | 2.23 (7.57) |
| **PosEmo** |  |  |  |  |  |  |  |  |  |  |  |  |  |
| Mean (SD) | 6.86 (13.81) | 7.16 (14.55) | 6.76 (13.97) | 6.94 (14.11) | 6.89 (14.10) | 7.17 (14.66) | 6.88 (14.22) | 6.79 (14.33) | 6.88 (14.37) | 6.80 (14.05) | 7.04 (14.47) | 7.10 (14.43) | 6.94 (14.27) |
| **Work** |  |  |  |  |  |  |  |  |  |  |  |  |  |
| Mean (SD) | 2.26 (5.79) | 2.34 (6.02) | 2.37 (5.93) | 2.27 (5.75) | 2.26 (5.90) | 2.14 (5.60) | 2.09 (5.83) | 2.13 (5.76) | 2.25 (5.68) | 2.23 (5.80) | 2.15 (5.78) | 1.98 (5.55) | 2.20 (5.78) |
| **Leisure** |  |  |  |  |  |  |  |  |  |  |  |  |  |
| Mean (SD) | 2.15 (5.90) | 1.95 (5.47) | 1.99 (5.52) | 2.11 (5.82) | 1.95 (5.54) | 1.91 (5.53) | 1.91 (5.60) | 2.02 (5.91) | 1.96 (5.74) | 1.94 (5.83) | 1.98 (5.93) | 2.18 (6.24) | 2.01 (5.78) |
| **Home** |  |  |  |  |  |  |  |  |  |  |  |  |  |
| Mean (SD) | 0.40 (2.27) | 0.36 (2.17) | 0.37 (2.29) | 0.38 (2.45) | 0.38 (2.41) | 0.37 (2.25) | 0.38 (2.46) | 0.34 (2.16) | 0.40 (2.85) | 0.45 (3.12) | 0.46 (3.25) | 0.45 (3.10) | 0.40 (2.64) |
| **Health** |  |  |  |  |  |  |  |  |  |  |  |  |  |
| Mean (SD) | 0.62 (3.44) | 0.58 (3.25) | 0.62 (3.13) | 0.58 (3.10) | 0.64 (3.41) | 0.58 (2.93) | 0.58 (3.38) | 0.57 (3.48) | 0.61 (3.39) | 0.61 (3.44) | 0.56 (3.02) | 0.57 (3.10) | 0.59 (3.26) |

Note*.* NegEmo = Negative emotion; PosEmo = positive emotion; Linguistic Inquiry and Word Count (LIWC) scores represent percentages of total in-category words within a given text.
