# Supplementary material for: Fluctuations of psychological states on Twitter before and during COVID-19
Source: PLoS One. 2022 Dec 14;17(12):e0278018. doi: 10.1371/journal.pone.0278018 (PMC9750014; doi:10.1371/journal.pone.0278018)
Supplement: S5 Table — Note. AIC = Akaike information criterion; BIC = Bayesian information criterion. (DOCX) [file pone.0278018.s005.docx]

**Table S5**

*Testing for fluctuations over the different months within a year, based on chi-square tests between the models with and without the factor month for London*

|  | **Model without factor month** | | | **Full model with factor month** | | | **Chi-square test** | |
| --- | --- | --- | --- | --- | --- | --- | --- | --- |
| *Construct (London)* | *AIC* | *BIC* | *Deviance* | *AIC* | *BIC* | *Deviance* | *Deviance change*  *(χ^2^)* | *Pr(>χ^2^)* |
| Sadness 2020 | 91483 | 91517 | 91475 | 91371 | 91497 | 91341 | 133.67 | <.001 |
| Sadness 2019 | 68911 | 68944 | 68903 | 68910 | 69034 | 68880 | 22.91 | .018 |
| Anxiety 2020 | 73710 | 73744 | 73702 | 73480 | 73605 | 73450 | 252.45 | <.001 |
| Anxiety 2019 | 54319 | 54352 | 54311 | 54327 | 54450 | 54297 | 14.78 | .193 |
| Anger 2020 | 93569 | 93603 | 93561 | 93438 | 93564 | 93408 | 153.23 | <.001 |
| Anger 2019 | 73504 | 73537 | 73496 | 73497 | 73621 | 73467 | 28.40 | .003 |
| Negative emotion 2020 | 142957 | 142990 | 142949 | 142903 | 143028 | 142873 | 75.92 | <.001 |
| Negative emotion 2019 | 115491 | 115524 | 115483 | 115472 | 115596 | 115442 | 40.84 | <.001 |
| Positive emotion 2020 | 192034 | 192068 | 192026 | 191922 | 192047 | 191892 | 134.59 | <.001 |
| Positive emotion 2019 | 161744 | 161777 | 161736 | 161712 | 161836 | 161682 | 53.87 | <.001 |
| Work 2020 | 162003 | 162037 | 161995 | 161815 | 161941 | 161785 | 210.3 | <.001 |
| Work 2019 | 133610 | 133643 | 133602 | 133561 | 133684 | 133531 | 71.82 | <.001 |
| Leisure 2020 | 149502 | 149535 | 149494 | 149338 | 149464 | 149308 | 185.21 | <.001 |
| Leisure 2019 | 125111 | 125144 | 125103 | 125069 | 125193 | 125039 | 63.75 | <.001 |
| Home 2020 | 92508 | 92541 | 92500 | 91814 | 91940 | 91784 | 715.55 | <.001 |
| Home 2019 | 69490 | 69523 | 69482 | 69468 | 69592 | 69438 | 43.43 | <.001 |
| Health 2020 | 104813 | 104847 | 104805 | 104299 | 104425 | 104269 | 536.49 | <.001 |
| Health 2019 | 80114 | 80147 | 80106 | 80111 | 80235 | 80081 | 25.68 | .007 |

Note. AIC = Akaike information criterion; BIC = Bayesian information criterion.
