# Supplementary material for: Fluctuations of psychological states on Twitter before and during COVID-19
Source: PLoS One. 2022 Dec 14;17(12):e0278018. doi: 10.1371/journal.pone.0278018 (PMC9750014; doi:10.1371/journal.pone.0278018)
Supplement: S6 Table — Note. AIC = Akaike information criterion; BIC = Bayesian information criterion. (DOCX) [file pone.0278018.s006.docx]

**Table S6**

*Testing for fluctuations over the different months within a year, based on chi-square tests between the models with and without the factor month for New York*

|  | **Model without factor month** | | | **Full model with factor month** | | | **Chi-square test** | |
| --- | --- | --- | --- | --- | --- | --- | --- | --- |
| *Construct (New York)* | *AIC* | *BIC* | *Deviance* | *AIC* | *BIC* | *Deviance* | *Deviance change*  *(χ^2^)* | *Pr(>χ^2^)* |
| Sadness 2020 | 57082 | 57114 | 57074 | 57038 | 57156 | 57008 | 66.73 | <.001 |
| Sadness 2019 | 40306 | 40337 | 40298 | 40314 | 40430 | 40284 | 14.1 | .228 |
| Anxiety 2020 | 45008 | 45040 | 45000 | 44915 | 45033 | 44885 | 115.59 | <.001 |
| Anxiety 2019 | 30474 | 30505 | 30466 | 30456 | 30571 | 30426 | 40.50 | <.001 |
| Anger 2020 | 66573 | 66604 | 66565 | 66256 | 66374 | 66226 | 339.39 | <.001 |
| Anger 2019 | 47596 | 47627 | 47588 | 47598 | 47713 | 47568 | 20.52 | .039 |
| Negative emotion 2020 | 91409 | 91441 | 91401 | 91300 | 91418 | 91270 | 130.84 | <.001 |
| Negative emotion 2019 | 68626 | 68657 | 68618 | 68611 | 68727 | 68581 | 36.86 | <.001 |
| Positive emotion 2020 | 114959 | 114990 | 114951 | 114865 | 114983 | 114835 | 116.4 | <.001 |
| Positive emotion 2019 | 90792 | 90823 | 90784 | 90774 | 90889 | 90744 | 40.25 | <.001 |
| Work 2020 | 97874 | 97906 | 97866 | 97796 | 97914 | 97766 | 100.89 | <.001 |
| Work 2019 | 76622 | 76653 | 76614 | 76591 | 76707 | 76561 | 53.12 | <.001 |
| Leisure 2020 | 90993 | 91024 | 90985 | 90804 | 90923 | 90774 | 210.49 | <.001 |
| Leisure 2019 | 73271 | 73302 | 73263 | 73254 | 73370 | 73224 | 38.81 | <.001 |
| Home 2020 | 57538 | 57569 | 57530 | 57147 | 57265 | 57117 | 412.44 | <.001 |
| Home 2019 | 41463 | 41494 | 41455 | 41469 | 41584 | 41439 | 16.63 | .119 |
| Health 2020 | 66893 | 66924 | 66885 | 66583 | 66701 | 66553 | 331.58 | <.001 |
| Health 2019 | 48650 | 48681 | 48642 | 48647 | 48763 | 48617 | 24.97 | .009 |

Note. AIC = Akaike information criterion; BIC = Bayesian information criterion.
