# Supplementary material for: Fluctuations of psychological states on Twitter before and during COVID-19
Source: PLoS One. 2022 Dec 14;17(12):e0278018. doi: 10.1371/journal.pone.0278018 (PMC9750014; doi:10.1371/journal.pone.0278018)
Supplement: S7 Table — Note. CI = confidence interval; ICC = intraclass correlation coefficient; LIWC = Linguistic Inquiry and Word Count; uid = user id; wc = word count. (DOCX) [file pone.0278018.s007.docx]

**Table S7**

*Mixed negative binomial regression models predicting the monthly number of words belonging to the LIWC dictionary “Sadness”*

|  | **Sadness London 2020** | | | **Sadness London 2019** | | | **Sadness New York 2020** | | | **Sadness New York 2019** | | |
| --- | --- | --- | --- | --- | --- | --- | --- | --- | --- | --- | --- | --- |
| *Predictor* | *Incidence rate ratios* | *95% CI* | *p* | *Incidence rate ratios* | *95% CI* | *p* | *Incidence rate ratios* | *95% CI* | *p* | *Incidence rate ratios* | *95% CI* | *p* |
| (Intercept) | 0.00 | 0.00 – 0.00 | <0.001 | 0.00 | 0.00 – 0.00 | <0.001 | 0.00 | 0.00 – 0.01 | <0.001 | 0.00 | 0.00 – 0.00 | <0.001 |
| month [February] | 1.02 | 0.97 – 1.07 | 0.527 | 1.07 | 1.003 – 1.13 | 0.038 | 0.94 | 0.88 – 1.001 | 0.056 | 1.09 | 1.01 – 1.18 | 0.026 |
| month [March] | 1.18 | 1.12 – 1.23 | <0.001 | 1.04 | 0.98 – 1.11 | 0.170 | 1.03 | 0.98 – 1.10 | 0.248 | 1.03 | 0.95 – 1.11 | 0.494 |
| month [April] | 1.17 | 1.12 – 1.22 | <0.001 | 1.05 | 0.99 – 1.12 | 0.087 | 1.11 | 1.05 – 1.18 | <0.001 | 1.06 | 0.98 – 1.15 | 0.150 |
| month [May] | 1.08 | 1.04 – 1.13 | 0.001 | 1.03 | 0.97 – 1.09 | 0.322 | 1.07 | 1.01 – 1.13 | 0.022 | 1.01 | 0.93 – 1.09 | 0.858 |
| month [June] | 1.05 | 1.003 – 1.10 | 0.036 | 1.04 | 0.98 – 1.10 | 0.196 | 1.00 | 0.95 – 1.06 | 0.919 | 1.07 | 0.98 – 1.15 | 0.117 |
| month [July] | 1.02 | 0.97 – 1.07 | 0.426 | 1.02 | 0.96 – 1.08 | 0.540 | 1.03 | 0.97 – 1.09 | 0.304 | 1.04 | 0.96 – 1.12 | 0.339 |
| month [August] | 1.04 | 0.99 – 1.09 | 0.082 | 1.05 | 0.99 – 1.12 | 0.080 | 1.07 | 1.01 – 1.14 | 0.018 | 1.05 | 0.97 – 1.14 | 0.223 |
| month [September] | 1.05 | 1.001 – 1.10 | 0.044 | 1.03 | 0.98 – 1.10 | 0.257 | 1.06 | 0.99 – 1.12 | 0.070 | 1.09 | 1.004 – 1.17 | 0.038 |
| month [October] | 1.05 | 1.01 – 1.10 | 0.028 | 1.08 | 1.02 – 1.14 | 0.009 | 1.05 | 0.99 – 1.11 | 0.087 | 1.03 | 0.95 – 1.11 | 0.441 |
| month [November] | 1.12 | 1.07 – 1.18 | <0.001 | 1.01 | 0.95 – 1.07 | 0.735 | 1.13 | 1.07 – 1.20 | <0.001 | 1.01 | 0.93 – 1.09 | 0.856 |
| month [December] | 1.13 | 1.08 – 1.19 | <0.001 | 1.11 | 1.04 – 1.17 | 0.001 | 1.09 | 1.03 – 1.16 | 0.002 | 1.08 | 1.0007 – 1.17 | 0.048 |
| wc [log] | 2.65 | 2.62 – 2.68 | <0.001 | 2.69 | 2.65 – 2.74 | <0.001 | 2.63 | 2.58 – 2.67 | <0.001 | 2.65 | 2.59 – 2.71 | <0.001 |
| **Random Effects** | | | | | | | | | | | | |
| σ^2^ | 0.71 | | | 0.91 | | | 0.67 | | | 0.90 | | |
| τ_00_ | 0.23 _uid_ | | | 0.28 _uid_ | | | 0.29 _uid_ | | | 0.33 _uid_ | | |
| ICC | 0.25 | | | 0.24 | | | 0.30 | | | 0.27 | | |
| N | 2942 _uid_ | | | 2724 _uid_ | | | 1788 _uid_ | | | 1609 _uid_ | | |
| Observations | 32097 | | | 28390 | | | 19330 | | | 16373 | | |
| Marginal *R*^2^ / Conditional *R*^2^ | 0.722 / 0.791 | | | 0.637 / 0.723 | | | 0.719 / 0.803 | | | 0.635 / 0.733 | | |

Note*.* CI = confidence interval; ICC = intraclass correlation coefficient; LIWC = Linguistic Inquiry and Word Count; uid = user id; wc = word count.
