# Supplementary material for: Fluctuations of psychological states on Twitter before and during COVID-19
Source: PLoS One. 2022 Dec 14;17(12):e0278018. doi: 10.1371/journal.pone.0278018 (PMC9750014; doi:10.1371/journal.pone.0278018)
Supplement: S8 Table — Note. CI = confidence interval; ICC = intraclass correlation coefficient; LIWC = Linguistic Inquiry and Word Count; uid = user id; wc = word count. (DOCX) [file pone.0278018.s008.docx]

**Table S8**

*Mixed negative binomial regression models predicting the monthly number of words belonging to the LIWC dictionary “Anxiety”*

|  | **Anxiety London 2020** | | | **Anxiety London 2019** | | | **Anxiety New York 2020** | | | **Anxiety New York 2019** | | |
| --- | --- | --- | --- | --- | --- | --- | --- | --- | --- | --- | --- | --- |
| *Predictor* | *Incidence rate ratios* | *95% CI* | *p* | *Incidence rate ratios* | *95% CI* | *p* | *Incidence rate ratios* | *95% CI* | *p* | *Incidence rate ratios* | *95% CI* | *p* |
| (Intercept) | 0.00 | 0.00 – 0.00 | <0.001 | 0.00 | 0.00 – 0.00 | <0.001 | 0.00 | 0.00 – 0.00 | <0.001 | 0.00 | 0.00 – 0.00 | <0.001 |
| month [February] | 1.03 | 0.97 – 1.09 | 0.342 | 1.00 | 0.92 – 1.07 | 0.926 | 1.09 | 1.01 – 1.18 | 0.032 | 0.98 | 0.88 – 1.08 | 0.625 |
| month [March] | 1.42 | 1.34 – 1.50 | <0.001 | 1.08 | 1.003 – 1.16 | 0.041 | 1.35 | 1.26 – 1.45 | <0.001 | 1.00 | 0.91 – 1.10 | 0.999 |
| month [April] | 1.12 | 1.06 – 1.18 | <0.001 | 0.98 | 0.91 – 1.06 | 0.646 | 1.09 | 1.02 – 1.18 | 0.016 | 0.95 | 0.86 – 1.05 | 0.305 |
| month [May] | 1.11 | 1.05 – 1.18 | <0.001 | 0.98 | 0.91 – 1.06 | 0.616 | 1.07 | 0.99 – 1.16 | 0.057 | 0.97 | 0.88 – 1.07 | 0.557 |
| month [June] | 1.12 | 1.06 – 1.18 | <0.001 | 1.01 | 0.94 – 1.09 | 0.756 | 1.14 | 1.05 – 1.22 | 0.001 | 0.89 | 0.81 – 0.99 | 0.027 |
| month [July] | 1.05 | 0.99 – 1.11 | 0.121 | 0.99 | 0.92 – 1.06 | 0.784 | 1.06 | 0.99 – 1.14 | 0.104 | 1.08 | 0.98 – 1.19 | 0.125 |
| month [August] | 1.08 | 1.02 – 1.14 | 0.010 | 0.99 | 0.92 – 1.06 | 0.789 | 1.08 | 0.99 – 1.16 | 0.053 | 1.03 | 0.93 – 1.13 | 0.554 |
| month [September] | 1.12 | 1.06 – 1.19 | <0.001 | 1.03 | 0.96 – 1.11 | 0.425 | 1.15 | 1.07 – 1.24 | <0.001 | 1.04 | 0.95 – 1.15 | 0.391 |
| month [October] | 1.14 | 1.08 – 1.21 | <0.001 | 1.05 | 0.98 – 1.13 | 0.180 | 1.11 | 1.03 – 1.19 | 0.007 | 1.11 | 1.01 – 1.22 | 0.026 |
| month [November] | 1.11 | 1.05 – 1.18 | <0.001 | 1.03 | 0.96 – 1.10 | 0.463 | 1.10 | 1.02 – 1.18 | 0.010 | 0.98 | 0.89 – 1.08 | 0.721 |
| month [December] | 1.09 | 1.03 – 1.16 | 0.003 | 1.03 | 0.96 – 1.11 | 0.421 | 1.00 | 0.93 – 1.08 | 0.988 | 0.91 | 0.83 – 1.01 | 0.068 |
| wc [log] | 2.90 | 2.86 – 2.95 | <0.001 | 2.88 | 2.83 – 2.94 | <0.001 | 2.86 | 2.80 – 2.92 | <0.001 | 2.87 | 2.79 – 2.95 | <0.001 |
| **Random Effects** | | | | | | | | | | | | |
| σ^2^ | 1.01 | | | 1.26 | | | 1.00 | | | 1.31 | | |
| τ_00_ | 0.29 _uid_ | | | 0.35 _uid_ | | | 0.31 _uid_ | | | 0.39 _uid_ | | |
| ICC | 0.22 | | | 0.22 | | | 0.24 | | | 0.23 | | |
| N | 2942 _uid_ | | | 2724 _uid_ | | | 1788 _uid_ | | | 1609 _uid_ | | |
| Observations | 32097 | | | 28390 | | | 19330 | | | 16373 | | |
| Marginal *R*^2^ / Conditional *R*^2^ | 0.693 / 0.762 | | | 0.599 / 0.687 | | | 0.689 / 0.762 | | | 0.597 / 0.689 | | |

Note*.* CI = confidence interval; ICC = intraclass correlation coefficient; LIWC = Linguistic Inquiry and Word Count; uid = user id; wc = word count.
