# Supplementary material for: Fluctuations of psychological states on Twitter before and during COVID-19
Source: PLoS One. 2022 Dec 14;17(12):e0278018. doi: 10.1371/journal.pone.0278018 (PMC9750014; doi:10.1371/journal.pone.0278018)
Supplement: S9 Table — Note. CI = confidence interval; ICC = intraclass correlation coefficient; LIWC = Linguistic Inquiry and Word Count; uid = user id; wc = word count. (DOCX) [file pone.0278018.s009.docx]

**Table S9**

*Mixed negative binomial regression models predicting the monthly number of words belonging to the LIWC dictionary “Anger”*

|  | **Anger London 2020** | | | **Anger London 2019** | | | **Anger New York 2020** | | | **Anger New York 2019** | | |
| --- | --- | --- | --- | --- | --- | --- | --- | --- | --- | --- | --- | --- |
| *Predictor* | *Incidence rate ratios* | *95% CI* | *p* | *Incidence rate ratios* | *95% CI* | *p* | *Incidence rate ratios* | *95% CI* | *p* | *Incidence rate ratios* | *95% CI* | *p* |
| (Intercept) | 0.00 | 0.00 – 0.00 | <0.001 | 0.00 | 0.00 – 0.00 | <0.001 | 0.00 | 0.00 – 0.01 | <0.001 | 0.00 | 0.00 – 0.00 | <0.001 |
| month [February] | 0.98 | 0.93 – 1.02 | 0.361 | 0.99 | 0.94 – 1.05 | 0.824 | 1.02 | 0.96 – 1.07 | 0.549 | 1.01 | 0.94 – 1.08 | 0.843 |
| month [March] | 0.89 | 0.85 – 0.92 | <0.001 | 1.03 | 0.98 – 1.09 | 0.236 | 0.93 | 0.88 – 0.98 | 0.004 | 0.98 | 0.92 – 1.05 | 0.588 |
| month [April] | 0.91 | 0.87 – 0.95 | <0.001 | 1.00 | 0.94 – 1.05 | 0.908 | 0.94 | 0.89 – 0.99 | 0.012 | 0.97 | 0.91 – 1.04 | 0.430 |
| month [May] | 0.98 | 0.94 – 1.03 | 0.426 | 0.95 | 0.90 – 1.004 | 0.070 | 1.09 | 1.04 – 1.14 | 0.001 | 0.96 | 0.90 – 1.02 | 0.189 |
| month [June] | 1.10 | 1.06 – 1.15 | <0.001 | 1.02 | 0.96 – 1.08 | 0.521 | 1.32 | 1.25 – 1.38 | <0.001 | 0.98 | 0.91 – 1.04 | 0.483 |
| month [July] | 0.97 | 0.93 – 1.02 | 0.215 | 0.98 | 0.93 – 1.04 | 0.564 | 1.01 | 0.97 – 1.07 | 0.562 | 1.01 | 0.94 – 1.07 | 0.869 |
| month [August] | 0.97 | 0.93 – 1.02 | 0.214 | 1.04 | 0.98 – 1.10 | 0.189 | 1.04 | 0.99 – 1.09 | 0.139 | 1.07 | 1.01 – 1.14 | 0.032 |
| month [September] | 0.99 | 0.94 – 1.03 | 0.504 | 0.99 | 0.94 – 1.05 | 0.757 | 1.04 | 0.99 – 1.09 | 0.124 | 0.99 | 0.93 – 1.05 | 0.707 |
| month [October] | 0.98 | 0.94 – 1.03 | 0.470 | 1.02 | 0.97 – 1.08 | 0.492 | 1.04 | 0.99 – 1.10 | 0.087 | 1.02 | 0.96 – 1.09 | 0.554 |
| month [November] | 0.96 | 0.92 – 1.002 | 0.064 | 1.00 | 0.95 – 1.06 | 0.913 | 1.02 | 0.97 – 1.07 | 0.552 | 0.99 | 0.93 – 1.05 | 0.709 |
| month [December] | 0.94 | 0.90 – 0.99 | 0.009 | 1.07 | 1.01 – 1.13 | 0.016 | 0.94 | 0.89 – 0.99 | 0.022 | 1.04 | 0.97 – 1.10 | 0.285 |
| wc [log] | 2.85 | 2.82 – 2.89 | <0.001 | 2.78 | 2.73 – 2.83 | <0.001 | 2.76 | 2.72 – 2.80 | <0.001 | 2.79 | 2.73 – 2.85 | <0.001 |
| **Random Effects** | | | | | | | | | | | | |
| σ^2^ | 0.76 | | | 0.91 | | | 0.56 | | | 0.76 | | |
| τ_00_ | 0.77 _uid_ | | | 0.85 _uid_ | | | 0.86 _uid_ | | | 0.93 _uid_ | | |
| ICC | 0.51 | | | 0.48 | | | 0.61 | | | 0.55 | | |
| N | 2942 _uid_ | | | 2724 _uid_ | | | 1788 _uid_ | | | 1609 _uid_ | | |
| Observations | 32097 | | | 28390 | | | 19330 | | | 16373 | | |
| Marginal *R*^2^ / Conditional *R*^2^ | 0.649 / 0.826 | | | 0.560 / 0.773 | | | 0.656 / 0.864 | | | 0.586 / 0.814 | | |

Note*.* CI = confidence interval; ICC = intraclass correlation coefficient; LIWC = Linguistic Inquiry and Word Count; uid = user id; wc = word count.
