# Supplementary material for: Fluctuations of psychological states on Twitter before and during COVID-19
Source: PLoS One. 2022 Dec 14;17(12):e0278018. doi: 10.1371/journal.pone.0278018 (PMC9750014; doi:10.1371/journal.pone.0278018)
Supplement: S10 Table — Note. CI = confidence interval; ICC = intraclass correlation coefficient; LIWC = Linguistic Inquiry and Word Count; uid = user id; wc = word count. (DOCX) [file pone.0278018.s010.docx]

**Table S10**

*Mixed negative binomial regression models predicting the monthly number of words belonging to the LIWC dictionary “NegEmo”*

|  | **NegEmo London 2020** | | | **NegEmo London 2019** | | | **NegEmo New York 2020** | | | **NegEmo New York 2019** | | |
| --- | --- | --- | --- | --- | --- | --- | --- | --- | --- | --- | --- | --- |
| *Predictor* | *Incidence rate ratios* | *95% CI* | *p* | *Incidence rate ratios* | *95% CI* | *p* | *Incidence rate ratios* | *95% CI* | *p* | *Incidence rate ratios* | *95% CI* | *p* |
| (Intercept) | 0.01 | 0.01 – 0.01 | <0.001 | 0.01 | 0.01 – 0.01 | <0.001 | 0.02 | 0.01 – 0.02 | <0.001 | 0.01 | 0.01 – 0.02 | <0.001 |
| month [February] | 1.01 | 0.98 – 1.04 | 0.581 | 1.02 | 0.98 – 1.05 | 0.340 | 0.99 | 0.96 – 1.02 | 0.583 | 1.00 | 0.96 – 1.04 | 0.898 |
| month [March] | 1.07 | 1.05 – 1.10 | <0.001 | 1.03 | 0.99 – 1.07 | 0.055 | 1.03 | 0.99 – 1.06 | 0.082 | 0.99 | 0.95 – 1.03 | 0.592 |
| month [April] | 1.03 | 1.01 – 1.06 | 0.012 | 1.01 | 0.98 – 1.04 | 0.592 | 1.02 | 0.99 – 1.05 | 0.247 | 0.98 | 0.94 – 1.02 | 0.403 |
| month [May] | 1.04 | 1.01 – 1.06 | 0.004 | 0.97 | 0.94 – 1.004 | 0.096 | 1.05 | 1.02 – 1.09 | 0.001 | 0.96 | 0.92 – 0.99 | 0.029 |
| month [June] | 1.08 | 1.06 – 1.11 | <0.001 | 1.02 | 0.98 – 1.05 | 0.318 | 1.13 | 1.10 – 1.17 | <0.001 | 0.98 | 0.94 – 1.02 | 0.394 |
| month [July] | 1.01 | 0.99 – 1.04 | 0.331 | 0.98 | 0.95 – 1.02 | 0.326 | 1.02 | 0.99 – 1.05 | 0.172 | 1.03 | 0.99 – 1.07 | 0.215 |
| month [August] | 1.02 | 0.99 – 1.05 | 0.067 | 1.02 | 0.99 – 1.05 | 0.204 | 1.04 | 1.01 – 1.07 | 0.020 | 1.04 | 1.002 – 1.09 | 0.036 |
| month [September] | 1.03 | 1.003 – 1.06 | 0.025 | 1.00 | 0.97 – 1.04 | 0.793 | 1.05 | 1.02 – 1.09 | 0.001 | 1.01 | 0.97 – 1.05 | 0.553 |
| month [October] | 1.04 | 1.02 – 1.07 | 0.001 | 1.04 | 1.003 – 1.07 | 0.031 | 1.03 | 1.002 – 1.07 | 0.034 | 1.03 | 0.99 – 1.08 | 0.091 |
| month [November] | 1.04 | 1.02 – 1.07 | 0.001 | 1.01 | 0.98 – 1.05 | 0.461 | 1.03 | 0.99 – 1.06 | 0.100 | 0.98 | 0.94 – 1.02 | 0.228 |
| month [December] | 1.03 | 0.99 – 1.05 | 0.064 | 1.05 | 1.02 – 1.08 | 0.002 | 0.98 | 0.95 – 1.02 | 0.327 | 1.01 | 0.97 – 1.05 | 0.577 |
| wc [log] | 2.78 | 2.76 – 2.81 | <0.001 | 2.75 | 2.72 – 2.78 | <0.001 | 2.73 | 2.71 – 2.76 | <0.001 | 2.75 | 2.71 – 2.78 | <0.001 |
| **Random Effects** | | | | | | | | | | | | |
| σ^2^ | 0.25 | | | 0.33 | | | 0.21 | | | 0.31 | | |
| τ_00_ | 0.33 _uid_ | | | 0.40 _uid_ | | | 0.39 _uid_ | | | 0.46 _uid_ | | |
| ICC | 0.57 | | | 0.55 | | | 0.65 | | | 0.60 | | |
| N | 2942 _uid_ | | | 2724 _uid_ | | | 1788 _uid_ | | | 1609 _uid_ | | |
| Observations | 32097 | | | 28390 | | | 19330 | | | 16373 | | |
| Marginal *R*^2^ / Conditional *R*^2^ | 0.822 / 0.923 | | | 0.749 / 0.886 | | | 0.814 / 0.935 | | | 0.751 / 0.900 | | |

Note*.* CI = confidence interval; ICC = intraclass correlation coefficient; LIWC = Linguistic Inquiry and Word Count; uid = user id; wc = word count.
