# Supplementary material for: Fluctuations of psychological states on Twitter before and during COVID-19
Source: PLoS One. 2022 Dec 14;17(12):e0278018. doi: 10.1371/journal.pone.0278018 (PMC9750014; doi:10.1371/journal.pone.0278018)
Supplement: S11 Table — Note. CI = confidence interval; ICC = intraclass correlation coefficient; LIWC = Linguistic Inquiry and Word Count; uid = user id; wc = word count. (DOCX) [file pone.0278018.s011.docx]

**Table S11**

*Mixed negative binomial regression models predicting the monthly number of words belonging to the LIWC dictionary “PosEmo”*

|  | **PosEmo London 2020** | | | **PosEmo London 2019** | | | **PosEmo New York 2020** | | | **PosEmo New York 2019** | | |
| --- | --- | --- | --- | --- | --- | --- | --- | --- | --- | --- | --- | --- |
| *Predictor* | *Incidence rate ratios* | *95% CI* | *p* | *Incidence rate ratios* | *95% CI* | *p* | *Incidence rate ratios* | *95% CI* | *p* | *Incidence rate ratios* | *95% CI* | *p* |
| (Intercept) | 0.08 | 0.08 – 0.08 | <0.001 | 0.08 | 0.08 – 0.08 | <0.001 | 0.07 | 0.07 – 0.07 | <0.001 | 0.07 | 0.07 – 0.07 | <0.001 |
| month [February] | 1.01 | 0.99 – 1.03 | 0.177 | 1.01 | 0.99 – 1.03 | 0.153 | 1.00 | 0.98 – 1.03 | 0.696 | 1.01 | 0.99 – 1.03 | 0.445 |
| month [March] | 0.99 | 0.97 – 1.003 | 0.116 | 1.01 | 0.99 – 1.03 | 0.120 | 0.97 | 0.95 – 0.99 | 0.010 | 0.97 | 0.95 – 0.99 | 0.021 |
| month [April] | 1.02 | 1.01 – 1.04 | 0.005 | 1.01 | 0.99 – 1.03 | 0.188 | 0.99 | 0.97 – 1.01 | 0.401 | 1.00 | 0.97 – 1.02 | 0.934 |
| month [May] | 0.98 | 0.97 – 0.99 | 0.030 | 1.04 | 1.02 – 1.06 | <0.001 | 0.97 | 0.95 – 0.99 | 0.016 | 1.00 | 0.97 – 1.02 | 0.728 |
| month [June] | 0.97 | 0.96 – 0.99 | 0.001 | 1.02 | 0.99 – 1.04 | 0.061 | 0.95 | 0.93 – 0.97 | <0.001 | 1.02 | 0.99 – 1.04 | 0.144 |
| month [July] | 0.99 | 0.97 – 1.004 | 0.166 | 1.02 | 1.0005 – 1.04 | 0.043 | 0.97 | 0.95 – 0.99 | 0.018 | 0.98 | 0.96 – 1.01 | 0.149 |
| month [August] | 0.97 | 0.96 – 0.99 | 0.001 | 0.99 | 0.98 – 1.01 | 0.577 | 0.98 | 0.96 – 0.99 | 0.030 | 0.98 | 0.96 – 1.003 | 0.099 |
| month [September] | 0.96 | 0.95 – 0.98 | <0.001 | 1.01 | 0.99 – 1.03 | 0.290 | 0.95 | 0.93 – 0.97 | <0.001 | 0.99 | 0.96 – 1.01 | 0.268 |
| month [October] | 0.97 | 0.96 – 0.99 | 0.001 | 1.01 | 0.99 – 1.03 | 0.419 | 0.97 | 0.95 – 0.99 | 0.004 | 0.98 | 0.96 – 1.01 | 0.165 |
| month [November] | 0.98 | 0.96 – 0.99 | 0.011 | 1.02 | 1.01 – 1.04 | 0.008 | 1.02 | 1.0006 – 1.04 | 0.043 | 1.01 | 0.99 – 1.03 | 0.447 |
| month [December] | 1.02 | 1.0008 – 1.03 | 0.039 | 1.04 | 1.03 – 1.06 | <0.001 | 1.02 | 0.99 – 1.04 | 0.169 | 1.02 | 0.99 – 1.05 | 0.075 |
| wc [log] | 2.54 | 2.53 – 2.55 | <0.001 | 2.55 | 2.53 – 2.56 | <0.001 | 2.55 | 2.53 – 2.57 | <0.001 | 2.55 | 2.53 – 2.57 | <0.001 |
| **Random Effects** | | | | | | | | | | | | |
| σ^2^ | 0.09 | | | 0.10 | | | 0.09 | | | 0.11 | | |
| τ_00_ | 0.16 _uid_ | | | 0.15 _uid_ | | | 0.17 _uid_ | | | 0.16 _uid_ | | |
| ICC | 0.64 | | | 0.60 | | | 0.65 | | | 0.59 | | |
| N | 2942 _uid_ | | | 2724 _uid_ | | | 1788 _uid_ | | | 1609 _uid_ | | |
| Observations | 32097 | | | 28390 | | | 19330 | | | 16373 | | |
| Marginal *R*^2^ / Conditional *R*^2^ | 0.899 / 0.964 | | | 0.881 / 0.952 | | | 0.898 / 0.964 | | | 0.881 / 0.951 | | |

Note*.* CI = confidence interval; ICC = intraclass correlation coefficient; LIWC = Linguistic Inquiry and Word Count; uid = user id; wc = word count.
