# Supplementary material for: Fluctuations of psychological states on Twitter before and during COVID-19
Source: PLoS One. 2022 Dec 14;17(12):e0278018. doi: 10.1371/journal.pone.0278018 (PMC9750014; doi:10.1371/journal.pone.0278018)
Supplement: S12 Table — Note. CI = confidence interval; ICC = intraclass correlation coefficient; LIWC = Linguistic Inquiry and Word Count; uid = user id; wc = word count. (DOCX) [file pone.0278018.s012.docx]

**Table S12**

*Mixed negative binomial regression models predicting the monthly number of words belonging to the LIWC dictionary “Work”*

|  | **Work London 2020** | | | **Work London 2019** | | | **Work New York 2020** | | | **Work New York 2019** | | |
| --- | --- | --- | --- | --- | --- | --- | --- | --- | --- | --- | --- | --- |
| *Predictor* | *Incidence rate ratios* | *95% CI* | *p* | *Incidence rate ratios* | *95% CI* | *p* | *Incidence rate ratios* | *95% CI* | *p* | *Incidence rate ratios* | *95% CI* | *p* |
| (Intercept) | 0.02 | 0.02 – 0.02 | <0.001 | 0.02 | 0.02 – 0.02 | <0.001 | 0.02 | 0.02 – 0.02 | <0.001 | 0.02 | 0.02 – 0.02 | <0.001 |
| month [February] | 1.02 | 0.99 – 1.05 | 0.141 | 1.01 | 0.98 – 1.03 | 0.702 | 1.03 | 0.99 – 1.06 | 0.076 | 1.01 | 0.97 – 1.05 | 0.663 |
| month [March] | 1.06 | 1.03 – 1.08 | <0.001 | 0.98 | 0.96 – 1.01 | 0.197 | 1.08 | 1.05 – 1.12 | <0.001 | 1.01 | 0.97 – 1.04 | 0.780 |
| month [April] | 0.96 | 0.93 – 0.98 | <0.001 | 1.00 | 0.97 – 1.03 | 0.961 | 1.03 | 1.001 – 1.07 | 0.042 | 0.98 | 0.94 – 1.02 | 0.246 |
| month [May] | 0.96 | 0.94 – 0.98 | 0.001 | 1.00 | 0.97 – 1.03 | 0.943 | 1.00 | 0.97 – 1.03 | 0.887 | 0.97 | 0.94 – 1.01 | 0.184 |
| month [June] | 0.99 | 0.97 – 1.02 | 0.559 | 0.97 | 0.95 – 1.0001 | 0.051 | 1.05 | 1.02 – 1.08 | 0.002 | 0.96 | 0.92 – 0.99 | 0.022 |
| month [July] | 0.98 | 0.96 – 1.01 | 0.144 | 0.98 | 0.95 – 1.01 | 0.142 | 1.03 | 1.0005 – 1.07 | 0.046 | 0.94 | 0.90 – 0.97 | 0.001 |
| month [August] | 0.97 | 0.95 – 0.99 | 0.042 | 0.96 | 0.93 – 0.98 | 0.001 | 1.02 | 0.98 – 1.05 | 0.347 | 0.97 | 0.93 – 1.004 | 0.085 |
| month [September] | 1.02 | 0.99 – 1.05 | 0.098 | 1.00 | 0.97 – 1.03 | 0.929 | 1.03 | 0.99 – 1.06 | 0.123 | 1.01 | 0.97 – 1.04 | 0.742 |
| month [October] | 0.99 | 0.97 – 1.02 | 0.551 | 0.99 | 0.96 – 1.02 | 0.397 | 0.97 | 0.94 – 1.004 | 0.098 | 0.99 | 0.96 – 1.03 | 0.697 |
| month [November] | 0.95 | 0.93 – 0.98 | <0.001 | 1.02 | 0.99 – 1.05 | 0.157 | 0.98 | 0.95 – 1.02 | 0.324 | 0.96 | 0.93 – 0.99 | 0.044 |
| month [December] | 0.91 | 0.89 – 0.94 | <0.001 | 0.93 | 0.91 – 0.96 | <0.001 | 0.96 | 0.93 – 0.99 | 0.024 | 0.92 | 0.89 – 0.96 | <0.001 |
| wc [log] | 2.74 | 2.72 – 2.76 | <0.001 | 2.72 | 2.70 – 2.75 | <0.001 | 2.78 | 2.75 – 2.81 | <0.001 | 2.78 | 2.74 – 2.81 | <0.001 |
| **Random Effects** | | | | | | | | | | | | |
| σ^2^ | 0.20 | | | 0.23 | | | 0.20 | | | 0.25 | | |
| τ_00_ | 0.31 _uid_ | | | 0.33 _uid_ | | | 0.36 _uid_ | | | 0.40 _uid_ | | |
| ICC | 0.61 | | | 0.60 | | | 0.65 | | | 0.62 | | |
| N | 2942 _uid_ | | | 2724 _uid_ | | | 1788 _uid_ | | | 1609 _uid_ | | |
| Observations | 32097 | | | 28390 | | | 19330 | | | 16373 | | |
| Marginal *R*^2^ / Conditional *R*^2^ | 0.837 / 0.937 | | | 0.794 / 0.916 | | | 0.830 / 0.940 | | | 0.784 / 0.918 | | |

Note*.* CI = confidence interval; ICC = intraclass correlation coefficient; LIWC = Linguistic Inquiry and Word Count; uid = user id; wc = word count.
