# Supplementary material for: Fluctuations of psychological states on Twitter before and during COVID-19
Source: PLoS One. 2022 Dec 14;17(12):e0278018. doi: 10.1371/journal.pone.0278018 (PMC9750014; doi:10.1371/journal.pone.0278018)
Supplement: S13 Table — Note. CI = confidence interval; ICC = intraclass correlation coefficient; LIWC = Linguistic Inquiry and Word Count; uid = user id; wc = word count. (DOCX) [file pone.0278018.s013.docx]

**Table S13**

*Mixed negative binomial regression models predicting the monthly number of words belonging to the LIWC dictionary “Leisure”*

|  | **Leisure London 2020** | | | **Leisure London 2019** | | | **Leisure New York 2020** | | | **Leisure New York 2019** | | |
| --- | --- | --- | --- | --- | --- | --- | --- | --- | --- | --- | --- | --- |
| *Predictor* | *Incidence rate ratios* | *95% CI* | *p* | *Incidence rate ratios* | *95% CI* | *p* | *Incidence rate ratios* | *95% CI* | *p* | *Incidence rate ratios* | *95% CI* | *p* |
| (Intercept) | 0.02 | 0.02 – 0.02 | <0.001 | 0.02 | 0.02 – 0.02 | <0.001 | 0.02 | 0.02 – 0.02 | <0.001 | 0.02 | 0.02 – 0.02 | <0.001 |
| month [February] | 1.00 | 0.97 – 1.03 | 0.858 | 1.01 | 0.98 – 1.04 | 0.592 | 0.99 | 0.95 – 1.03 | 0.612 | 0.98 | 0.94 – 1.02 | 0.285 |
| month [March] | 0.99 | 0.96 – 1.02 | 0.480 | 1.00 | 0.97 – 1.03 | 0.796 | 0.94 | 0.91 – 0.98 | 0.001 | 1.00 | 0.96 – 1.04 | 0.968 |
| month [April] | 1.00 | 0.97 – 1.03 | 0.880 | 1.06 | 1.03 – 1.10 | <0.001 | 0.91 | 0.88 – 0.95 | <0.001 | 1.03 | 0.99 – 1.08 | 0.126 |
| month [May] | 0.97 | 0.94 – 0.99 | 0.014 | 1.06 | 1.03 – 1.09 | <0.001 | 0.91 | 0.88 – 0.94 | <0.001 | 1.00 | 0.96 – 1.04 | 0.972 |
| month [June] | 0.92 | 0.89 – 0.94 | <0.001 | 1.05 | 1.02 – 1.08 | 0.002 | 0.82 | 0.79 – 0.85 | <0.001 | 0.98 | 0.94 – 1.03 | 0.427 |
| month [July] | 1.07 | 1.04 – 1.10 | <0.001 | 1.03 | 0.99 – 1.06 | 0.086 | 0.94 | 0.90 – 0.97 | 0.001 | 1.00 | 0.96 – 1.04 | 0.976 |
| month [August] | 0.97 | 0.94 – 0.99 | 0.017 | 1.06 | 1.03 – 1.10 | <0.001 | 0.92 | 0.89 – 0.95 | <0.001 | 1.01 | 0.97 – 1.05 | 0.672 |
| month [September] | 0.94 | 0.92 – 0.97 | <0.001 | 0.99 | 0.96 – 1.02 | 0.494 | 0.92 | 0.89 – 0.96 | <0.001 | 0.99 | 0.95 – 1.03 | 0.674 |
| month [October] | 0.96 | 0.94 – 0.99 | 0.011 | 1.01 | 0.98 – 1.05 | 0.353 | 0.89 | 0.86 – 0.92 | <0.001 | 0.97 | 0.93 – 1.01 | 0.108 |
| month [November] | 0.93 | 0.91 – 0.96 | <0.001 | 1.01 | 0.98 – 1.04 | 0.577 | 0.92 | 0.89 – 0.96 | <0.001 | 0.99 | 0.95 – 1.03 | 0.546 |
| month [December] | 0.98 | 0.96 – 1.01 | 0.244 | 1.01 | 0.98 – 1.05 | 0.378 | 1.02 | 0.98 – 1.06 | 0.360 | 1.07 | 1.03 – 1.11 | 0.001 |
| wc [log] | 2.58 | 2.56 – 2.60 | <0.001 | 2.60 | 2.57 – 2.62 | <0.001 | 2.57 | 2.54 – 2.60 | <0.001 | 2.61 | 2.58 – 2.65 | <0.001 |
| **Random Effects** | | | | | | | | | | | | |
| σ^2^ | 0.26 | | | 0.28 | | | 0.26 | | | 0.29 | | |
| τ_00_ | 0.24 _uid_ | | | 0.24 _uid_ | | | 0.27 _uid_ | | | 0.29 _uid_ | | |
| ICC | 0.48 | | | 0.46 | | | 0.51 | | | 0.50 | | |
| N | 2942 _uid_ | | | 2724 _uid_ | | | 1788 _uid_ | | | 1609 _uid_ | | |
| Observations | 32097 | | | 28390 | | | 19330 | | | 16373 | | |
| Marginal *R*^2^ / Conditional *R*^2^ | 0.823 / 0.908 | | | 0.789 / 0.885 | | | 0.816 / 0.909 | | | 0.782 / 0.890 | | |

Note*.* CI = confidence interval; ICC = intraclass correlation coefficient; LIWC = Linguistic Inquiry and Word Count; uid = user id; wc = word count.
