# Supplementary material for: Fluctuations of psychological states on Twitter before and during COVID-19
Source: PLoS One. 2022 Dec 14;17(12):e0278018. doi: 10.1371/journal.pone.0278018 (PMC9750014; doi:10.1371/journal.pone.0278018)
Supplement: S14 Table — Note. CI = confidence interval; ICC = intraclass correlation coefficient; LIWC = Linguistic Inquiry and Word Count; uid = user id; wc = word count. (DOCX) [file pone.0278018.s014.docx]

**Table S14**

*Mixed negative binomial regression models predicting the monthly number of words belonging to the LIWC dictionary “Home”*

|  | **Home London 2020** | | | **Home London 2019** | | | **Home New York 2020** | | | **Home New York 2019** | | |
| --- | --- | --- | --- | --- | --- | --- | --- | --- | --- | --- | --- | --- |
| *Predictor* | *Incidence rate ratios* | *95% CI* | *p* | *Incidence rate ratios* | *95% CI* | *p* | *Incidence rate ratios* | *95% CI* | *p* | *Incidence rate ratios* | *95% CI* | *p* |
| (Intercept) | 0.00 | 0.00 – 0.00 | <0.001 | 0.00 | 0.00 – 0.00 | <0.001 | 0.00 | 0.00 – 0.00 | <0.001 | 0.00 | 0.00 – 0.00 | <0.001 |
| month [February] | 1.02 | 0.96 – 1.07 | 0.601 | 1.04 | 0.97 – 1.11 | 0.289 | 0.99 | 0.92 – 1.06 | 0.743 | 0.92 | 0.85 – 1.003 | 0.062 |
| month [March] | 1.52 | 1.44 – 1.60 | <0.001 | 0.98 | 0.91 – 1.04 | 0.463 | 1.43 | 1.34 – 1.52 | <0.001 | 0.91 | 0.83 – 0.99 | 0.023 |
| month [April] | 1.48 | 1.41 – 1.56 | <0.001 | 1.07 | 1.01 – 1.15 | 0.032 | 1.36 | 1.28 – 1.45 | <0.001 | 0.90 | 0.83 – 0.98 | 0.020 |
| month [May] | 1.30 | 1.24 – 1.37 | <0.001 | 1.02 | 0.95 – 1.09 | 0.590 | 1.12 | 1.05 – 1.20 | <0.001 | 0.92 | 0.84 – 0.99 | 0.042 |
| month [June] | 1.05 | 0.99 – 1.10 | 0.104 | 1.07 | 1.01 – 1.14 | 0.032 | 0.95 | 0.89 – 1.01 | 0.095 | 0.91 | 0.84 – 0.99 | 0.037 |
| month [July] | 1.10 | 1.04 – 1.16 | 0.001 | 1.10 | 1.03 – 1.17 | 0.003 | 1.05 | 0.98 – 1.12 | 0.171 | 0.91 | 0.84 – 0.99 | 0.038 |
| month [August] | 1.12 | 1.06 – 1.19 | <0.001 | 1.08 | 1.01 – 1.15 | 0.022 | 1.05 | 0.99 – 1.12 | 0.117 | 0.91 | 0.84 – 0.99 | 0.030 |
| month [September] | 1.10 | 1.04 – 1.17 | <0.001 | 1.03 | 0.97 – 1.10 | 0.329 | 0.97 | 0.91 – 1.03 | 0.347 | 0.92 | 0.84 – 0.99 | 0.036 |
| month [October] | 1.08 | 1.02 – 1.14 | 0.008 | 1.04 | 0.97 – 1.10 | 0.277 | 1.00 | 0.94 – 1.07 | 0.982 | 0.95 | 0.88 – 1.03 | 0.241 |
| month [November] | 1.07 | 1.02 – 1.14 | 0.009 | 1.01 | 0.95 – 1.08 | 0.766 | 1.04 | 0.98 – 1.11 | 0.197 | 0.95 | 0.87 – 1.03 | 0.197 |
| month [December] | 1.11 | 1.05 – 1.17 | <0.001 | 1.14 | 1.07 – 1.21 | <0.001 | 1.10 | 1.03 – 1.18 | 0.004 | 1.00 | 0.92 – 1.09 | 0.974 |
| wc [log] | 2.64 | 2.61 – 2.68 | <0.001 | 2.62 | 2.58 – 2.67 | <0.001 | 2.63 | 2.59 – 2.68 | <0.001 | 2.72 | 2.65 – 2.78 | <0.001 |
| **Random Effects** | | | | | | | | | | | | |
| σ^2^ | 0.76 | | | 0.95 | | | 0.71 | | | 0.94 | | |
| τ_00_ | 0.31 _uid_ | | | 0.33 _uid_ | | | 0.31 _uid_ | | | 0.39 _uid_ | | |
| ICC | 0.29 | | | 0.26 | | | 0.31 | | | 0.29 | | |
| N | 2942 _uid_ | | | 2724 _uid_ | | | 1788 _uid_ | | | 1609 _uid_ | | |
| Observations | 32097 | | | 28390 | | | 19330 | | | 16373 | | |
| Marginal *R*^2^ / Conditional *R*^2^ | 0.699 / 0.787 | | | 0.609 / 0.710 | | | 0.709 / 0.798 | | | 0.632 / 0.739 | | |

Note*.* CI = confidence interval; ICC = intraclass correlation coefficient; LIWC = Linguistic Inquiry and Word Count; uid = user id; wc = word count.
