# Supplementary material for: Fluctuations of psychological states on Twitter before and during COVID-19
Source: PLoS One. 2022 Dec 14;17(12):e0278018. doi: 10.1371/journal.pone.0278018 (PMC9750014; doi:10.1371/journal.pone.0278018)
Supplement: S15 Table — Note. CI = confidence interval; ICC = intraclass correlation coefficient; LIWC = Linguistic Inquiry and Word Count; uid = user id; wc = word count. (DOCX) [file pone.0278018.s015.docx]

**Table S15**

*Mixed negative binomial regression models predicting the monthly number of words belonging to the LIWC dictionary “Health”*

|  | **Health London 2020** | | | **Health London 2019** | | | **Health New York 2020** | | | **Health New York 2019** | | |
| --- | --- | --- | --- | --- | --- | --- | --- | --- | --- | --- | --- | --- |
| *Predictor* | *Incidence rate ratios* | *95% CI* | *p* | *Incidence rate ratios* | *95% CI* | *p* | *Incidence rate ratios* | *95% CI* | *p* | *Incidence rate ratios* | *95% CI* | *p* |
| (Intercept) | 0.00 | 0.00 – 0.00 | <0.001 | 0.00 | 0.00 – 0.01 | <0.001 | 0.01 | 0.01 – 0.01 | <0.001 | 0.01 | 0.01 – 0.01 | <0.001 |
| month [February] | 1.00 | 0.96 – 1.05 | 0.856 | 0.93 | 0.88 – 0.99 | 0.017 | 1.02 | 0.96 – 1.08 | 0.505 | 0.95 | 0.89 – 1.02 | 0.186 |
| month [March] | 1.35 | 1.29 – 1.40 | <0.001 | 0.91 | 0.86 – 0.97 | 0.001 | 1.33 | 1.26 – 1.41 | <0.001 | 1.04 | 0.97 – 1.11 | 0.225 |
| month [April] | 1.30 | 1.24 – 1.35 | <0.001 | 0.93 | 0.88 – 0.98 | 0.011 | 1.21 | 1.15 – 1.28 | <0.001 | 0.95 | 0.89 – 1.02 | 0.162 |
| month [May] | 1.18 | 1.13 – 1.23 | <0.001 | 0.95 | 0.90 – 1.01 | 0.088 | 1.13 | 1.07 – 1.20 | <0.001 | 1.05 | 0.98 – 1.12 | 0.193 |
| month [June] | 1.08 | 1.03 – 1.12 | 0.001 | 0.90 | 0.85 – 0.95 | <0.001 | 1.03 | 0.98 – 1.09 | 0.274 | 0.99 | 0.92 – 1.06 | 0.713 |
| month [July] | 1.07 | 1.02 – 1.12 | 0.003 | 0.90 | 0.85 – 0.95 | <0.001 | 1.01 | 0.96 – 1.07 | 0.716 | 0.95 | 0.89 – 1.01 | 0.124 |
| month [August] | 1.01 | 0.97 – 1.06 | 0.606 | 0.93 | 0.88 – 0.98 | 0.011 | 1.02 | 0.97 – 1.08 | 0.472 | 0.93 | 0.87 – 0.99 | 0.037 |
| month [September] | 1.08 | 1.03 – 1.13 | 0.001 | 0.91 | 0.86 – 0.96 | 0.001 | 1.01 | 0.96 – 1.07 | 0.611 | 1.00 | 0.93 – 1.07 | 0.979 |
| month [October] | 1.05 | 1.0003 – 1.09 | 0.048 | 0.94 | 0.89 – 0.99 | 0.017 | 1.03 | 0.97 – 1.09 | 0.326 | 1.00 | 0.94 – 1.07 | 0.982 |
| month [November] | 0.99 | 0.95 – 1.04 | 0.764 | 0.91 | 0.86 – 0.96 | 0.001 | 0.94 | 0.89 – 0.99 | 0.027 | 0.99 | 0.92 – 1.05 | 0.694 |
| month [December] | 1.04 | 0.99 – 1.08 | 0.120 | 0.90 | 0.86 – 0.95 | <0.001 | 0.96 | 0.91 – 1.02 | 0.188 | 0.99 | 0.92 – 1.06 | 0.729 |
| wc [log] | 2.76 | 2.72 – 2.79 | <0.001 | 2.70 | 2.66 – 2.75 | <0.001 | 2.70 | 2.66 – 2.75 | <0.001 | 2.64 | 2.59 – 2.70 | <0.001 |
| **Random Effects** | | | | | | | | | | | | |
| σ^2^ | 0.58 | | | 0.77 | | | 0.53 | | | 0.70 | | |
| τ_00_ | 0.33 _uid_ | | | 0.40 _uid_ | | | 0.28 _uid_ | | | 0.36 _uid_ | | |
| ICC | 0.36 | | | 0.34 | | | 0.34 | | | 0.34 | | |
| N | 2942 _uid_ | | | 2724 _uid_ | | | 1788 _uid_ | | | 1609 _uid_ | | |
| Observations | 32097 | | | 28390 | | | 19330 | | | 16373 | | |
| Marginal *R*^2^ / Conditional *R*^2^ | 0.747 / 0.837 | | | 0.644 / 0.765 | | | 0.762 / 0.844 | | | 0.670 / 0.782 | | |

Note*.* CI = confidence interval; ICC = intraclass correlation coefficient; LIWC = Linguistic Inquiry and Word Count; uid = user id; wc = word count.
