# Supplementary material for: Fluctuations of psychological states on Twitter before and during COVID-19
Source: PLoS One. 2022 Dec 14;17(12):e0278018. doi: 10.1371/journal.pone.0278018 (PMC9750014; doi:10.1371/journal.pone.0278018)
Supplement: S1 Fig — Note. Orange dotted lines show the beginnings of the first (London and New York) and second (London only) lockdown; purple dotted lines show the Linguistic Inquiry and Word Count (LIWC) reference values for tweets; LIWC scores represent percentages of total in-category words; monthly means are from the original sample; error bars denote bootstrapped 95% confidence intervals aggregated from 10,000 iterations. (DOCX) [file pone.0278018.s016.docx]

**Figure S1a**

*Monthly LIWC scores with bootstrapped 95% confidence intervals*


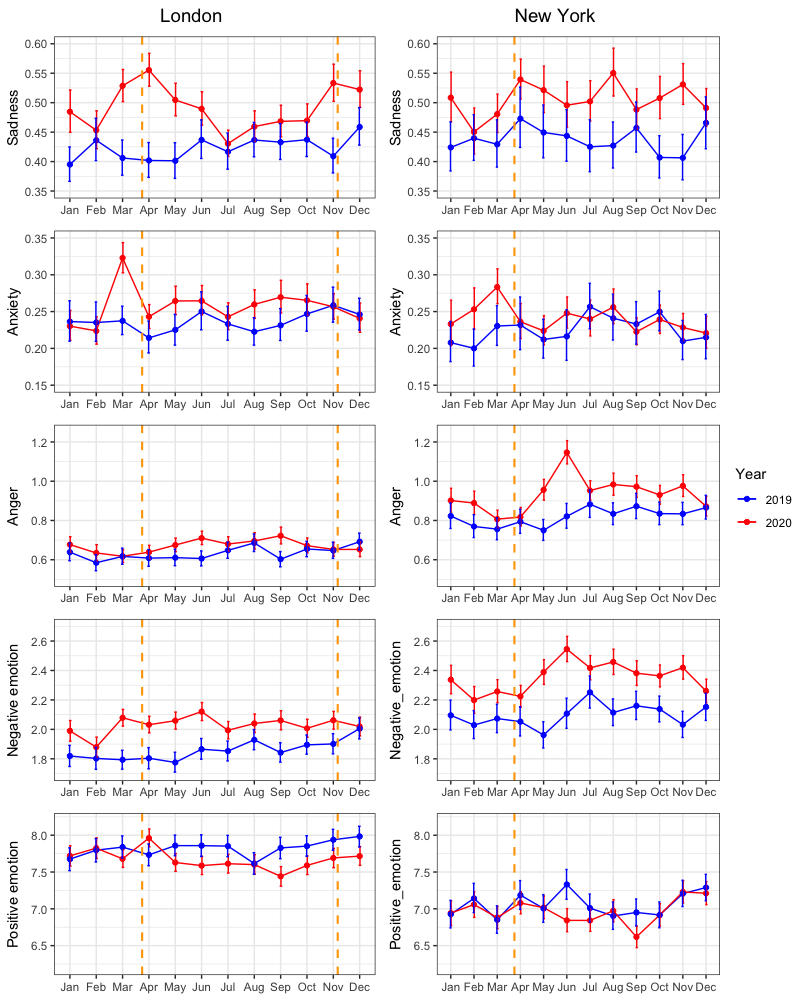


*Note.* Orange dotted lines show the beginnings of the first (London and New York) and second (London only) lockdown; purple dotted lines show the Linguistic Inquiry and Word Count (LIWC) reference values for tweets; LIWC scores represent percentages of total in-category words; monthly means are from the original sample; error bars denote bootstrapped 95% confidence intervals aggregated from 10,000 iterations.

**Figure S1b**

*Monthly LIWC scores with bootstrapped 95% confidence intervals*


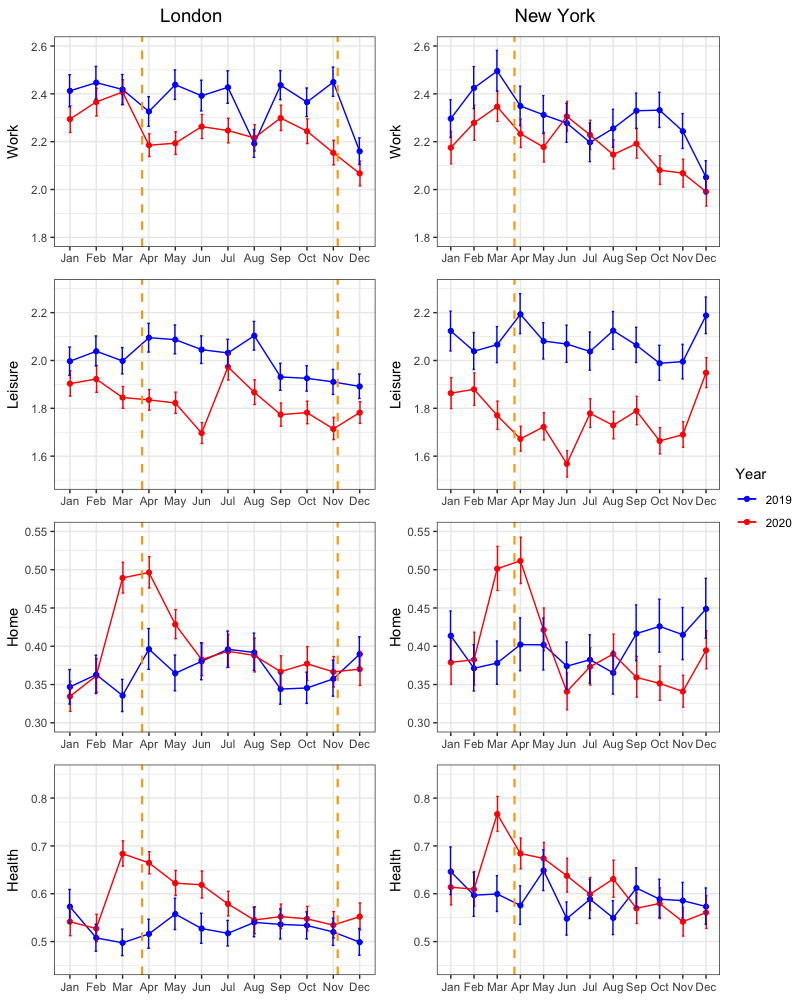


*Note.* Orange dotted lines show the beginnings of the first (London and New York) and second (London only) lockdown; purple dotted lines show the Linguistic Inquiry and Word Count (LIWC) reference values for tweets; LIWC scores represent percentages of total in-category words; monthly means are from the original sample; error bars denote bootstrapped 95% confidence intervals aggregated from 10,000 iterations.
