# Supplementary material for: Fluctuations of psychological states on Twitter before and during COVID-19
Source: PLoS One. 2022 Dec 14;17(12):e0278018. doi: 10.1371/journal.pone.0278018 (PMC9750014; doi:10.1371/journal.pone.0278018)
Supplement: S2 Fig — Note. Orange dotted lines show the beginnings of the first (London and New York) and second (London only) lockdown; neg = negative; pos = positive; neu = neutral; VADER = Valence Aware Dictionary for Sentiment Reasoning. (DOCX) [file pone.0278018.s017.docx]

**Figure S2**

*Equally weighted 29-day moving averages of VADER scores*


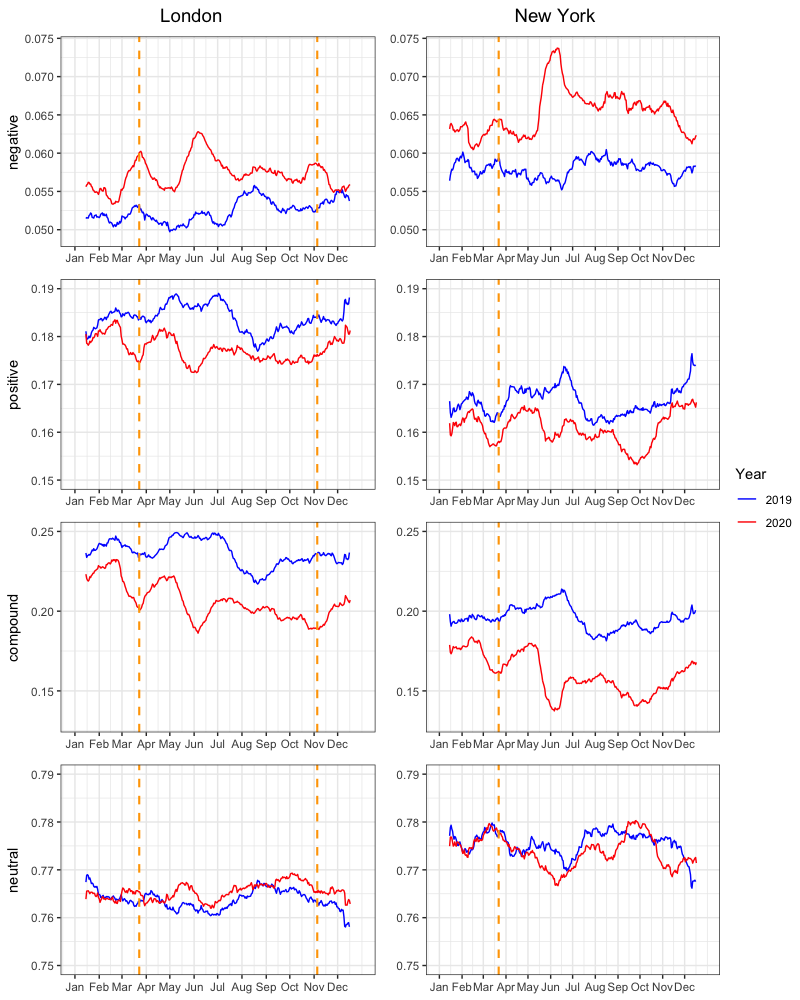


*Note.* Orange dotted lines show the beginnings of the first (London and New York) and second (London only) lockdown; neg = negative; pos = positive; neu = neutral; VADER = Valence Aware Dictionary for Sentiment Reasoning.
